# *Trypanosoma cruzi*: Seroprevalence Detection in Suburban Population of Santiago de Querétaro (Mexico)

**DOI:** 10.1100/2012/914129

**Published:** 2012-05-03

**Authors:** Ángeles López-Céspedes, Elena Villagrán, Kervin Briceño Álvarez, José Antonio de Diego, Hebert Luís Hernández-Montiel, Carlos Saldaña, Manuel Sānchez-Moreno, Clotilde Marín

**Affiliations:** ^1^Departamento de Parasitología, Facultad de Ciencias, Universidad de Granada, Severo Ochoa s/n, 18071 Granada, Spain; ^2^Departamento de Investigación Biomédica, Facultad de Medicina, UAQ, 76050 Santiago de Queretaro, QRO, Mexico; ^3^Unidad de Parasitología y Medicina Tropical, Departamento de Medicina Preventiva y Salud Pública, Facultad de Medicina, UAM, 28049 Madrid, Spain

## Abstract

*Objectives*. To evaluate the potential of iron-oxide dismutase excreted (SODeCRU) by *T. cruzi* as the antigen fraction in the serodiagnosis of Chagas disease and compile new epidemiological data on the seroprevalence of this disease in the suburban population of the city of Santiago de Querétaro (Mexico). *Design and Methods*. 258 human sera were analyzed by the techniques of ELISA and Western blot and using the homogenate and the SODeCRU. *Results*. A total of 31 sera were positive against ELISA/SODeCRU (12.4%), while 30 sera proved positive by WB/SODeCRU (11.6%). The comparison between the technique of ELISA and WB showed a sensitivity of 93%, and a specificity of 99%. The positive predictive value was 93% and the negative predictive value was 99%, with a Kappa (*κ*) value of 1. *Conclusions*. These preliminary data reveal the degree of infection of nonrural areas of Mexico and demonstrated that SODeCRU is an antigen useful to diagnose Chagas disease.

## 1. Introduction

Chagas disease, or American trypanosomiasis, is an infectious tropical disease caused by the blood flagellate *Trypanosoma cruzi*, which in its natural form is transmitted by Hemipteran vectors colloquially called “vinchucas” in Mexico and certain areas of South America. In addition, other forms of infection are known: blood transfusions, organ transplants, oral transmission, and congenital transmission. The first phase of the infection (acute phase) has clinical symptoms such as fever, discomfort, and cephalea and thus can be confused with the flu. These symptoms remit spontaneously, when the parasite passes to the mononuclear phagocytic system, nervous system (autonomic and myoenteric plexus), and the myocardium. Thereafter, years or even decades may pass without clinical manifestations (indeterminate phase). This phase can be diagnosed only by serological diagnosis. When the chronic phase appears (anatomical alterations, megaesophagus, megacolon, and especially myocardiopathy), the disease has no cure and most patients die a sudden death without the causes being known [1].

The Pan American Health Organization estimates that there are currently 7.7 million people infected by *T. cruzi* in 21 endemic countries, with the appearance of 41,200 cases per year, and 14,400 children are born annually with congenital Chagas disease. The countries most affected are Bolivia (6.8% of the cases reported), Argentina (4.1%), El Salvador (3.4%), Honduras (3.1%), Paraguay (2.5%), Guatemala (2%), Ecuador (1.7%), French Guyana, Guyana, and Surinam (1.2%), Venezuela (1.2%), Nicaragua (1.1%), Brazil (1%), and Mexico (1.3%) [2].

The enormous progress made in the control of Chagas disease in the last few decades indicates clearly that the obstacles against the complete elimination of transmission of *T. cruzi* to the human are mainly economic and political. In this context, there are additional noteworthy advances such as the more detailed understanding of the pathogen of Chagas disease, genetic analyses, new diagnostic techniques, and advances in the development of vaccines [3].

The diagnosis of Chagas disease depends on the phase of the disease. In the acute phase, the diagnosis is made by direct examination of the parasite in body fluids. In this acute or reactivation [4] phase, when the parasitaemia is high, the live parasite can be easily detected with its rapid movements. Other techniques used for the direct observation of the parasite include blood or droplet smear and exodiagnosis. These methods present, respectively, a sensitivity of 60–70% [5]. Despite being the most commonly used for their low costs, these techniques do not present high sensitivity and thus new inexpensive, quick methods are being sought [6, 7].

In the detection of Chagas disease, the PCR technique can be used, although in addition to the high cost and not being available in most of the laboratories of the endemic areas, it has highly variable sensitivity (45%–96.5%) and can amplify nonspecific products, giving false positives [5, 8, 9].

For all these reasons, the serological tests could represent the effective diagnosis for their sensitivity and relatively low cost. These are divided into two groups: conventional tests, which use the total parasite extract as the antigen, or soluble extract of an antigen complex; nonconventional tests, which usually use recombinant antigens or synthetic peptides [10]. Serological diagnosis gives different results according to the type of antigen used, the phase of the disease, and the type of immunoglobulins (IgG or IgM). The choice of the antigen is important for good results [11].

Many studies made to define a specific *T. cruzi* antigen that would increase the specificity of the serodiagnosis. One possibility is the iron-superoxide dismutase excreted by *T*. *cruzi *(Fe-SODeCRU or SODeCRU), which, in previous studies, has proven strongly immunogenic and highly specific, becoming useful to diagnose this disease [12, 13].

A prime objective of this study is to evaluate the potential of Fe-SODe of *T. cruzi *as the antigen fraction, in the diagnosis of Chagas disease, through the analysis of 258 human sera from the suburban area of the city of Santiago de Querétaro (Mexico), by the ELISA and Western Blot techniques. The standardization of the ELISA and Western blot techniques for screening Chagas patients, using the homogenate fraction and the SODeCRU as the antigen fraction, enables us to work towards the second objective: to compile epidemiological data on the seroprevalence of Chagas disease in suburban communities of the city of Santiago de Querétaro, which until now has not been studied.

## 2. Material and Methods

### 2.1. Parasites and Culture

Epimastigotes of *T. cruzi *(MHOM/ME/2006/H-4) were grown in axenic medium Trypanosomes Liquid Medium (MTL, Gibco) supplemented with 10% heat-inactivated foetal bovine serum at 28°C in Falcon flasks [14].

### 2.2. Donor Selection and Study Design

The sample was taken in the suburban areas (communities) of the city of Santiago Querétao (in the state of Querétaro, Mexico). The city is located at an average altitude of 1,820 m a.s.l., at 20° 35′ 34.8′′ of latitude north and 100° 23′ 31.6′′ of longitude west, a 221 km to the north-northwest of Mexico City. According to a June 2010 census (XIII Censo de Población y Vivienda), the population of the city centre was 626,517, with 801,833 inhabitants in the greater city, and 1,096,978 in the entire metropolitan zone, making it the 10th largest city in Mexico. 

The average annual temperature is 26.4°C, the maximum average temperature being around 37°C, in May, and the minimum temperature is 11.5°C, in January. The mean annual precipitation of the state is 638.3, with rains in summer from June to October (National Weather Service, http://smn.cna.gob.mx/).

A total of 258 human sera were evaluated (collected from February to October 2010), which were not grouped under any classification category (only numbered from 1 to 258). The sera were considered only as belonging to suburban communities pertaining to the city of Santiago de Querétaro, where the dwellings built under conditions of poverty occupy areas of hills and grazing areas, and domestic animals were present. The following analytic data were recorded: pathological alterations, age, sex, and place of origin of the patients (Table 1). The donors signed an informed consent form, and the study design was approved by the Ethics Committee for Research of the University of Granada (Spain).

A sample of 5 mL of whole blood was drawn from the ulnar vein of each human into assay tubes (Vacuttainer, Beckton-Dickinson, USA) and kept at 4°C. The negative control sera (20, healthy or asymptomatic human, who had never received a blood transfusion, nor organ transplant, nor had lived in a country endemic of Chagas disease) were obtained by the health services in Granada (Spain), which were not reactive to the Western Blot techniques.

### 2.3. Total Extract of the Parasite (Fraction H)

The parasite culture (in the exponential growth phase) was concentrated by centrifugation at 1500 rpm for 10 min. The pellet of the cells was washed twice and resuspended in ice-cold STE buffer (0.25 M sucrose, 25 mM Tris-HCl, 1 mM EDTA, pH 7.8) (Buffer 1). Afterwards, the pellet was suspended (0.5–0.6 g wet weight mL^−1^) in 3 mL of buffer 1 and disrupted by three cycles of sonic disintegration, 30 s each at 60 V. The sonicated homogenate was centrifuged at 1500 rpm for 10 min at 4°C, and the pellet was washed three times with buffer 1 for a total supernatant fraction of 9 mL. This fraction was centrifuged (2500 rpm for 10 min at 4°C), and the supernatant (fraction H) was collected [12].

### 2.4. Extraction and Purification of the SOD Excreted (SODeCRU)

Parasite forms in the exponential growth phase, obtained as described above, were concentrated by centrifugation at 1500 rpm for 10 min, the pellet of the cells was washed twice in MTL medium without serum, and the number of cells was counted in a haemocytometric chamber and distributed into aliquots of 5 × 10^9^ parasites/mL. Afterwards, the parasites were again grown in MTL medium without serum for 24 h; the supernatant was collected by centrifugation at 1500 rpm for 10 min and then passed through a filter of 0.45-*μ*m pore size, and solid ammonium sulphate added. The protein fraction, which precipitated at between 35 and 85% salt concentration, was centrifuged (9000 rpm for 20 min at 4°C), redissolved in 2.5 mL of 20 mM potassium phosphate buffer (pH 7.8) containing 1 mM EDTA (Buffer 2), and dialysed in a Sephadex G-25 column (Pharmacia, PD 10), previously balanced with Buffer 2, bringing it to a final volume of 2.5 mL (fraction SODeCRU) [16].

Both fractions, H and SODeCRU, were used as antigen fractions in the ELISA and Western Blot assays. The protein content was determined using the Bio-Rad test, based on the Bradford method (Sigma Immunochemical, St. Louis), with bovine serum albumin as a standard [16].

### 2.5. Serological Assay (ELISA)

For the ELISA assay, H and SODeCRU of the parasites, cultured and processed as described above, were used as the antigen fraction in all cases. The total homogenate (fraction H) and purified protein fraction (SODeCRU) at a concentration of 5 and 1.5 *μ*g, respectively, was coated onto polystyrene microtitre plates (Nunc, Denmark) in carbonate buffer (pH 8.2) for 2 h at 37°C. The antigen remaining on the plate was eliminated by washing three times with PBS-Tween 20 0.05% (washing buffer). Free adsorption sites were taken by incubation (2 h at 37°C) with blocking buffer (PBS-Tween 20 0.2%, BSA 1%). After being washed as described previously, the plates were incubated (45 min at 37°C) with serum dilution of 1 : 100 in washing buffer. After a second washing, the plates were incubated in darkness for 20 min with 100 *μ*L of an enzyme-conjugated antibody (Anti-IgG human peroxidase, Sigma) at a dilution of 1 : 1000. The enzyme reaction was developed with the chromogenic substrate OPD (o-Phenylenediamine dihydrochloride, Sigma) and 10 *μ*L of 30% H_2_O_2_ per 25 mL for 20 min in the dark. The reaction was stopped by addition of 50 *μ*L of HCl 3N. Absorbance was read at 492 nm in a microplate reader (Sunrise, TECAN). All the samples were analysed in triplicate in polystyrene microtitre plates. Mean and standard deviations (SD) of the optical densities of the negative control sera (20 healthy humans) were used to calculate the cut-off value (mean + 3 × SD) [12].

### 2.6. Western Blot Analysis

The antigen fraction of SODe-CRU (at a concentration 1.5 *μ*g of protein) was run on IEF 3–9 gels and afterwards transferred to nitrocellulose membrane (Hybond C Extra, Amersham Pharmacia Biotech ) using the Phast-Transfer kit, as described by the manufacturer (Phast-System handbook). The membrane was blocked for 2 h at room temperature using 0.4% gelatine and 0.2% Tween 20 in PBS, followed by three washes in 0.1% Tween 20 in PBS (PBS-T), and incubated for 2 h at room temperature, with donor sera at a dilution of 1/100. Before being washed, the membrane was further incubated for 2 h at room temperature with the second antibody, anti-human immunoglobulin G (Fc specific) peroxidase conjugate (Sigma Immunochemical; dilution 1/1,000). After washing as above, the substrate diaminobenzidine (0.5 mg/mL in buffer Tris/HCl 0.1 M, pH 7.4, containing 1/5000 H_2_O_2_ [10 v/v]) was added and the reaction stopped with several washes in distilled water [12].

## 3. Results and Discussion

Due to the high incidence of Chagas disease in a vast variety of endemic areas and given the risk involved for humans as well as other species of mammals that act as natural reservoirs of *T. cruzi*, it is of utmost importance that the populations at high risk be provided with prevention measures. Everyday it becomes more necessary to develop a highly sensitive and specific diagnostic method to offer the adequate treatment in the least time possible to reduce the transmission of this parasitosis.

**Figure 1:**
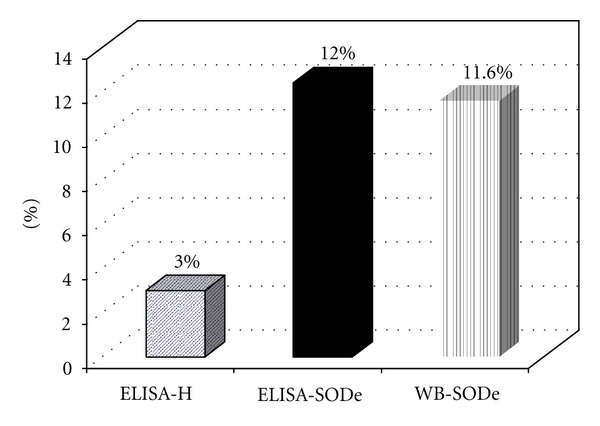
Seroprevalence of Chagas disease in suburban population of Querétaro city (Mexico) by ELISA (Homogenate and SODe) and Western Blot SODe tests.

In this sense, the present study proposes the use of SODeCRU as a highly effective antigen to diagnose the infections by *T. cruzi* in human populations living in endemic zones of the disease. The different homogenate fractions (H) and SODeCRU of *T. cruzi* obtained through the methodology described in Materials and Methods were tested by ELISA, against 258 human sera from suburban zones of the city of Santiago de Querétaro (Mexico). Of the total analysed, 8 sera proved positive against the H antigen fraction of *T. cruzi* (ELISA/(H)), for a prevalence of 3.0% (Figure 1).

For an evaluation of the antigenic potential of SODeCRU and its possible use in the immunodiagnosis of Chagas disease, these same sera were tested by the techniques of ELISA/SODeCRU and WB/SODeCRU. In this case, 31 proved positive against ELISA/SODeCRU from *T. cruzi* (for a prevalence of 12.0%), while 30 sera were positive for WB/SODeCRU (prevalence of 11.6%) (Figure 1).

**Figure 2:**
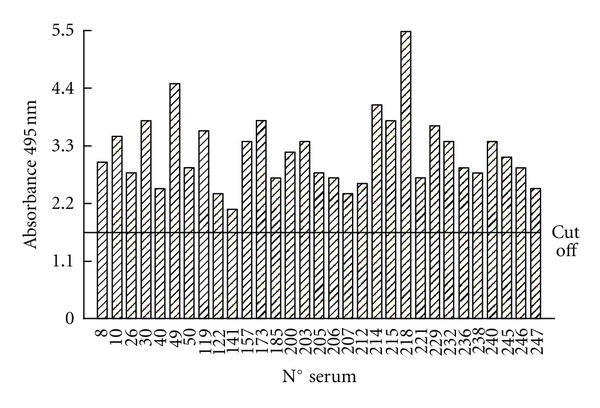
Results of the assay of 258 serum samples from human, collected in the suburban population of Querétaro city (Mexico) by enzyme-linked immunosorbent assay using the Fe-SODe by *T. cruzi*, as antigen at a dilution of 1/100. The mean and standard deviation of the optical densities of the control sera were used to calculate the cut-off value (mean × 3_standard deviation).

**Figure 3:**
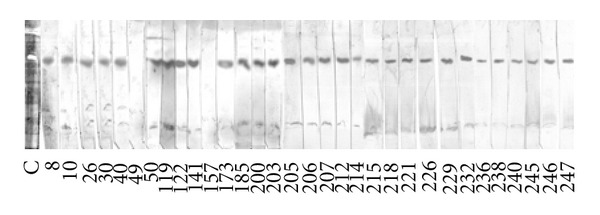
Immunoblot of the positive sera of the 258 human sera collected in the suburban population of Querétaro city (Mexico) against the SODe antigen from *T. cruzi* epimastigotes at a serum dilution of 1/100. Line C: SODe activity in isoelectrofocus and staining following the technique of Beyer and Fridovich [15].

The agreement between the results for ELISA/SODeCRU and WB/SODeCRU was almost 100%, with only the sera 49 and 157 showing positive with ELISA/SODeCRU but negative for WB/Fe-SODe, and, conversely, serum 226 gave a positive with WB/SODeCRU but negative with ELISA/SODeCRU (Figures 2 and 3). This minor discrepancy indicated that the ELISA/SODeCRU can give false positives, whereas the WB detects lower antigen concentrations. However, the ELISA/SODeCRU continued to present high sensitivity and specificity. In addition, due to the low cost with respect to the WB, it can be considered a more adequate tool to use in rural endemic areas, where an adequate health system is not available to provide the equipment necessary to run the high-cost diagnostic tests [16].

 Also, the presence of false positives in the ELISA technique may be due to cross reactions with other protozoa, fundamentally with *Trypanosoma rangeli* and with species belonging to the species belonging to the genus *Leishmania*, among others. This is a serious drawback in patients from geographical areas harbouring other infectious agents. However, in previous works, we have demonstrated that Fe-SODe is species specific and thus does not present cross reactions with other trypanosomatids [12, 14, 17].

The main challenge that health workers face when undertaking an epidemiological study of Chagas disease is the choice of the diagnostic method. Numerous authors support the choice of serological tests [11, 18, 19], while others suggest that serological methods are adequate only when combined with additional ones, this implying the use of two different techniques to be compared [20].

All parasitic protozoa investigated to data have been found to possess superoxide dismutase (SOD) linked to iron (Fe), this enzyme being considered a virulence factor, permitting the invading parasites to survive the oxidant offensive activated by the host [21]. In addition, studies made with parasites belonging to the family *Trypanosomatidae* have corroborated the presence of this Fe-SODe [17, 22]. The differences between the protozoan Fe-SODe and those of the hosts, together with the immunogenic properties, make this Fe-SODe useful as a feasible molecular marker for the development of new diagnostic methods, in addition to potential therapeutic targets for designing new anti-Chagas drugs with the potential of taking advantage of the structural differences between Fe-SOD protozoans and the copper-zinc-SOD of most eukaryotes [22].

Evaluating the results and taking into account the total of the human sera analysed with the ELISA/SODeCRU technique and comparing it to the technique of WB/SODeCRU as golden standard test, we found that the sensitivity of this technique reached 93%, with specificity of 99% (Table 2). The predictive value for positives with the ELISA/SODeCRU technique was 93%, and the predictive value for negatives was 99%. The Kappa (*k)* index of 1 confirmed that the proportion of agreement was total beyond random, between both assays ELISA/SODeCRU and WB/SODeCRU.

In general, because of the few cases documented parasitologically, Chagas disease is thought to be uncommon in Mexico, although it is probable that, like other diseases, cases are underrecorded for the lack of adequate diagnosis. The cases reported in the country correspond to the states of de Oaxaca, Chiapas, Jalisco, Michoacán, Guerrero, Zacatecas, Yucatán, Veracruz, Federal District of México, Sonora, and San Luis Potosí [23, 24]. The prevalence reported is extremely varied, the data going from 2.8% for the state of Nuevo León, to 16.8% reported for the state of Veracruz [25, 26]. In the state of Querétaro, the prevalence of this disease is unknown and data are available only for the year 2005 [17], when a prevalence of 8.6% was found in the rural population. For this reason, the second objective of the present study was to provide new epidemiological data on the disease in this state of Mexico, focusing on the suburban areas of the capital of this state.

**Figure 4:**
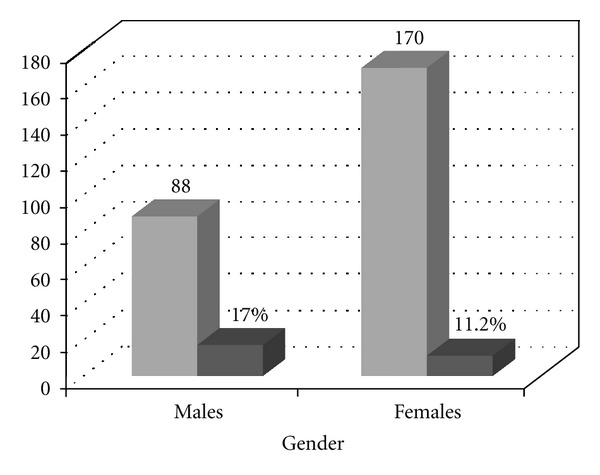
Seroprevalence of Chagas disease distributed by genders compared with the total of sera. Total samples analysed 258 (88 males and 170 females).

**Figure 5:**
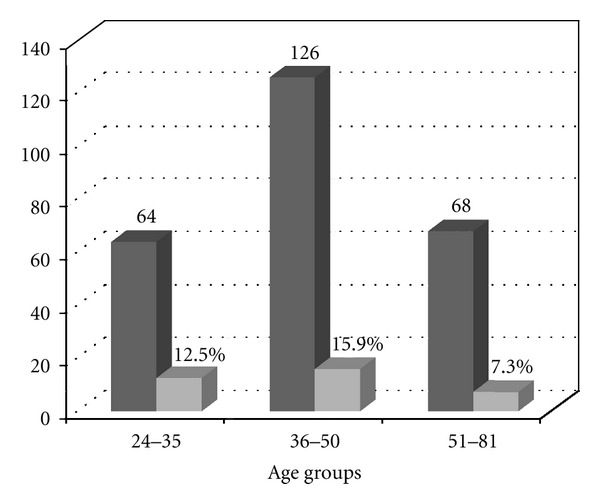
Seroprevalence of Chagas disease distributed by age groups compared with the total of sera. For range from 24 to 35, *n* = 64, range from 36 to 50,  *n* = 126, and range from 51 to 81, *n* = 68.

The prevalence found in the present study is significantly greater than that reported in 2005. This could be due to a transfer of the disease from the rural setting to the urban one, and, on the other hand, it could be due to an increase in the disease. Prevalence in males was higher than in females (17.0% versus 11.2%, resp.) (Figure 4), in agreement with findings reported by other authors [27], although, in some cases [28], the differences are not so significant. These discrepancies might be the result of cultural, behavioural, and socioeconomic differences among regions. The prevalence by ages shows that the group of persons between 36 and 50 years presented the highest prevalence (15.9%; Figure 5). The individuals that tested positive in our study presented analytical alterations, some of them compatible with the chronic phase of the disease.

**Figure 6:**
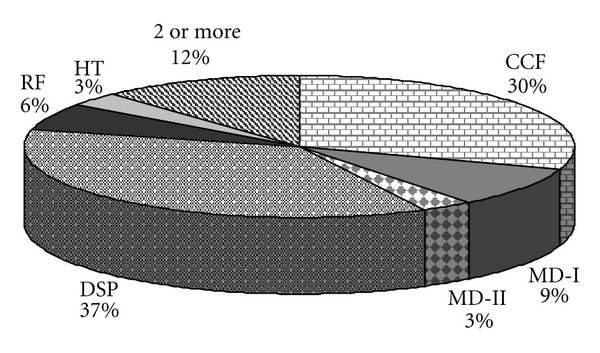
Associated pathologies in positive patients of Chagas Disease. CCF: chronic cardiac failure; RF: renal failure; HT: hyperthyroidism; DSP: dyslipidemia; MD-I: mellitus diabetes type 1; MD-II: mellitus diabetes type 2; 2 or more represents the combination of two or more of these pathologies.

Some 35% of the patients presented some type of dyslipidaemia (DSP), diabetes mellitus types I and II, acute kidney failure followed by chronic heart failure, the latter reaching 29% of the interviewees, who presented high values of cardiac enzymes (CK-MB; Figure 6).

 It was demonstrated that Fe-SOD excreted by *T. cruzi* was highly immunogenic and of high specificity, making it useful to diagnose Chagas disease at the same time as it provided epidemiological data on the seroprevalence of this disease in the suburban population of Santiago de Querétaro, formerly unstudied. These preliminary data reveal the degree of infection of nonrural areas of Mexico.

## Figures and Tables

**Table 1:** Relationship of positive sera of human from Queretaro (Mexico) by ELISA and Western Blot against different antigen fractions of *Trypanosoma cruzi* associated with age, gender, and pathology.

Sera	ELISA HOG^(a)^	ELISA SODe^ (b)^	WB SODe^(c)^	Age	Gender	Pathology^(d)^
8	−	+	+	53	M	DM-I
10	−	+	+	40	F	HPG
26	−	+	+	53	F	DM-I + ICC
30	−	+	+	43	M	CCF
40	−	+	+	32	F	HPC
49	+	+	−	43	M	ICC + DSP
50	+	+	+	39	F	CCF
119	+	+	+	45	F	HPG
122	+	+	+	35	M	DM-II
133	−	−	+	80	M	DM-II
141	−	+	+	27	F	RF
157	−	+	−	37	F	DSP
173	−	+	−	67	M	DSP
185	+	+	+	33	F	DSP
200	−	+	+	50	F	DSP
203	+	+	+	48	M	HPC
204	+	−	−	54	F	DM-I + DSP
205	−	+	+	43	F	CCF
206	−	+	+	27	F	DSP
207	−	+	+	29	F	CCF
212	−	+	+	48	M	HT
214	−	+	+	35	M	DSP
215	−	+	+	27	M	DSP
218	−	+	+	39	M	DM-I
221	−	+	+	40	F	HPC
226	−	−	+	37	M	CCF
229	−	+	+	51	F	CCF
232	−	+	+	42	F	DM-I
236	−	+	+	44	M	HPG
238	−	+	+	49	M	CCF
240	+	+	+	43	M	CCF
245	−	+	+	43	F	CCF
246	−	+	+	44	F	CCF
247	−	+	+	43	F	RF

^(a)^ELISA-HOG: Enzyme-Linked Immunosorbent Assay (ELISA) using total parasite extract (HOG) of *T. cruzi* as antigen fraction.

^(b)^ELISA-SODe: Enzyme-Linked Immunosorbent Assay (ELISA) using excreted superoxide dismutase (SODe) by epimastigotes of *T. cruzi* as antigen fraction.

^(c)^WB-SODe: Western Blot (WB) using excreted superoxide dismutase (SODe) by epimastigotes of *T. cruzi* as antigen fraction.

^(d)^Pathology abreviations: MD-I: mellitus diabetes type 1; MD-II: mellitus diabetes type 2; HPG: hipertrigliceridemia; CCF: chronic cardiac failure; HPC: hypercholesterolemia; DSP: dyslipidemia; RF: renal failure; HT: hyperthyroidism.

**Table 2:** Evaluation of the reliability to detect *Trypanosoma cruzi* antibodies, using ELISA technique with SODeCRU antigen, in 258 human sera from the suburban population in the city of Santiago de Querétaro (Mexico). The values given are estimated with Western Blot as the comparator.

	ELISA-SODeCRU
Sensitivity	93%
Specificity	99%
Positive predictive value	93%
Negative predictive value	99%
Kappa index	1
